# Anosmia in the course of COVID-19: A case report

**DOI:** 10.1097/MD.0000000000021280

**Published:** 2020-07-31

**Authors:** Neisa Santos Carvalho Alves Pissurno, Gislene Garcia de Castro Lichs, Evelyn Jaqueline Lima dos Santos, Angelita Fernandes Druzian, Sandra Maria do Valle Leone de Oliveira, Anamaria Mello Miranda Paniago

**Affiliations:** a Graduate Program in Infectious and Parasitic Diseases of Federal University of Mato Grosso do Sul; b Laboratório Central de Saúde Pública, Secretaria de Estado de Saúde; c Maria Aparecida Pedrossian University Hospital; d School of Medicine at Federal University of Mato Grosso do Sul, Campo Grande, Mato Grosso do Sul, Brazil.

**Keywords:** anos ia, case report, coronavirus disease, olfactory dysfunction, severe acute respiratory syndrome coronavirus 2

## Abstract

**Rationale:**

A sudden onset of anosmia has been recently recognized as a symptom of coronavirus disease (COVID-19).

**Patient concerns:**

Here, we describe a case of complete anosmia in a young male with COVID-19. Although he had fever and odynophagia, no abnormalities were observed in his nasopharyngeal mucosa, suggesting that his anosmia resulted from olfactory neuropathy.

**Diagnoses:**

COVID-19 was confirmed by RNA detection in nasopharyngeal swab specimen.

**Interventions:**

The patient received olfactory training and B complex vitamins.

**Outcomes:**

On day 30, the patient reported complete recovery of his sense of smell.

**Lessons:**

As early diagnosis is fundamental to control the spread of COVID-19 infection, we emphasize that anosmia identified in febrile cases during the COVID-19 epidemic may be a symptom indicative of the disease. Moreover, COVID-19-related anosmia can be completely reversible.

## 1 Introduction

The severe acute respiratory syndrome coronavirus 2 (SARS-CoV-2) is a new coronavirus that is highly contagious and responsible for the ongoing pandemic disease called coronavirus disease (COVID-19). Although the disease was first identified in December 2019, the World Health Organization did not declare a pandemic until March 11, 2020. Although individuals infected with SARS-CoV-2 may be asymptomatic, the disease may also present as a mild upper respiratory tract illness; however, many patients experience severe viral pneumonia that leads to respiratory failure, and, in many cases, death. Until April 20, 2020, 2,314,621 confirmed cases and 157,847 deaths were reported around the world.^[1]^

The main way to control the spread of COVID-19 is to prevent human-to-human transmission, which can be achieved through a combination of public health measures, including the rapid identification and isolation of infected people.^[1]^ A COVID-19 diagnosis is confirmed by viral RNA detection in nasopharyngeal swab specimens; nonetheless, in most of countries, COVID-19 tests are not available for screening and are only used to diagnose severe cases.

Diagnostic suspicion is based on nonspecific symptoms, such as fever, odynophagia, headache, and dry cough,^[2]^ which are present in almost all acute respiratory virus cases. Anosmia, which may be associated with the loss of taste, has been observed in European cases and seems to be a more specific symptom of COVID-19. Thus, during the pandemic, individuals with these symptoms should be tested for COVID-19; when tests are not available, isolation of the patient should be indicated.^[3]^

It should also be noted that olfactory dysfunction significantly influences the physical well-being, quality of life, safety, and nutritional status of those affected and becomes a greater problem when permanent.^[4]^ Yet very little is known about olfactory dysfunction in COVID-19. To contribute to existing knowledge and clinical evolution, and to reinforce the importance of this manifestation in the diagnosis and control of the disease, we report the particularities of a case that occurred at the beginning of the epidemic in Brazil.

## 2 Case report

**Figure 1:**
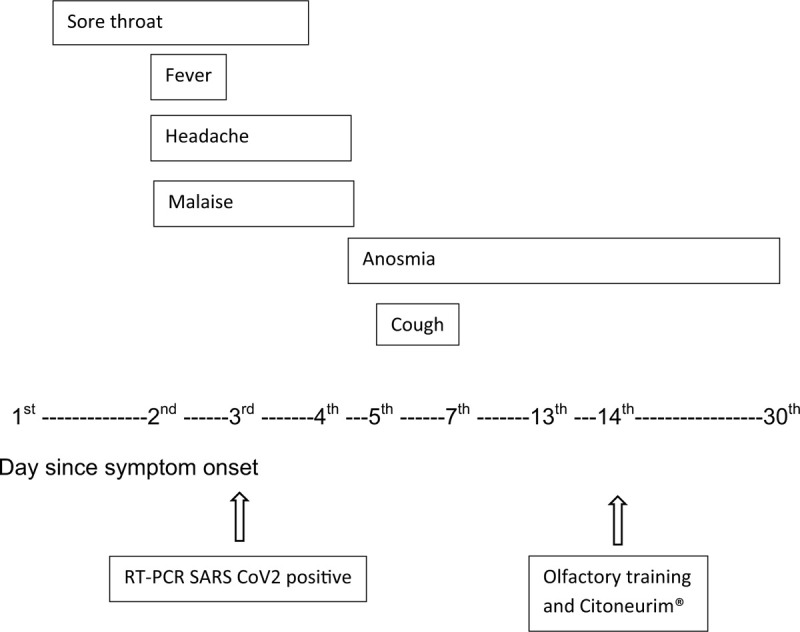
Timeline of events during the course of coronavirus disease.

Our patient was a 29-year-old male who traveled to Brazil from Europe (through Barcelona and Amsterdam) at the beginning of the COVID-19 pandemic in Europe and America (March 13, 2020). The patient was previously healthy and had no relevant medical history. He started with a sore throat on March 14, 2020. On day 2, the patient experienced fever (axillary temperature of 37.9° C), headache, and malaise (Fig. 1). He took 500 mg of dipyrone to reduce his fever and headache on day 2 and 3 of the disease, respectively. As he met the criteria of a suspect case definition by Brazilian standards, he was tested for SARS-CoV-2 on the third day. After a reverse transcriptase polymerase chain reaction assay was performed in accordance with the Berlin protocol,^[5]^ his results were positive.

On day 4, the patient's symptoms improved; however, he suddenly experienced complete anosmia. Yet, he did not experience nasal obstruction or coryza and no abnormalities were observed in his nasopharyngeal mucosa during the medical examination. On day 5, he complained of mild dry cough that persisted for 2 days (Fig. 1).

On day 7, the patient's symptoms disappeared, except for anosmia. As he was undergoing home quarantine, no complementary exams were performed. On day 13 of the disease, he noticed a small improvement in his sense of smell (Fig. 1).

From day 3 to day 13 of the disease course, no medication was prescribed to the patient.

On day 14, the patient began olfactory training, which consisted of inhaling identified bottles containing coffee, cinnamon, cloves, and lavender for 10 minutes a day. He also started taking 5000 IU of Citoneurin (Vitamin B1 [thiamine hydrochloride], 100 mg; Vitamin B6 [pyridoxine hydrochloride], 100 mg; Vitamin B12 [cyanocobalamin], 5000 mcg) a day orally. On day 30, the patient reported complete recuperation of his sense of smell (Fig. 1).

Since, no repeat testing for SARS-CoV-2 was performed, during follow-up, it was difficult to predict the duration of virus carriage.

The patient who is the subject of this report provided written informed consent for the details of his case to be published.

## 3 Discussion

Herein, we describe a case of a COVID-19 patient that presented with anosmia but did not experience any nasopharyngeal mucosa abnormalities during the course of the disease. Although olfactory dysfunction has been reported as a rare manifestation of many viruses,^[6]^ including severe acute respiratory syndrome during the 2003 epidemic,^[7]^ more recently, anosmia has been identified in many cases reported in Europe during the COVID-19 pandemic.^[3]^

Our patient experienced mild symptoms and was only tested for SARS-CoV-2 because he traveled to Europe during the epidemic. In this time, Barcelona and Amsterdam were cities with a large number of cases. The patient already experienced anosmia before his test results were obtained. After a few weeks, publications warned about the occurrence of olfactory and taste disorders in COVID-19 cases^[3,8]^ – symptoms which have been confirmed by this study. Our patient was very distressed as the media constantly reported COVID-19 deaths. Furthermore, the patient was worried that anosmia would remain as a sequela to COVID-19.^[6]^

Although females have been significantly more affected by olfactory and gustatory dysfunctions than males during the COVID-19 epidemic,^[3]^ our patient was a male. Moreover, the olfactory dysfunction appeared after he experienced fever and a sore throat. However, it is important to note that in some patients, this symptom may appear before the general or respiratory symptoms appear.^[3]^

Examinations to rule out other causes of olfactory dysfunction, such as magnetic resonance imaging of the brain, could not be performed in this patient because he was following the medical recommendation to self-isolate at home. Medical examination revealed no abnormalities in the oral or nasopharyngeal mucosa, and the patient had no history of significant disease. Furthermore, the only drug he used was dipyrone, which does not cause olfactory dysfunction.

As noted in this study, anosmia in COVID-19 cases usually has a sudden onset and is a temporary symptom that presents without other nasal symptoms such as obstruction or rhinorrhea.^[3,9]^ The olfactory system has a particular ability to regenerate throughout life, owing to stem cells that line the nasal cavity epithelium. However, olfactory receptors may lose their ability to regenerate due to age and pathological processes that lead to olfactory sensorineural dysfunction, resulting in partial (hyposmia) or complete (anosmia) loss of the sense of smell. It is believed that, by analogy to other viral infections of the upper airways, SARS-CoV-2 causes a sensorineural dysfunction in the sense of smell; nonetheless, its pathophysiological mechanism is still unclear. Angiotensin-converting enzyme 2 was recently identified as the receptor for SARS-CoV-2. This enzyme is present in many human organs, including the peripheral nervous system cells which may be harmed by SARS-CoV-2 through direct or indirect mechanisms.^[10]^

In addition to anosmia and ageusia, other peripheral nervous system symptoms have been reported in COVID-19 cases, such as visual impairment and neuralgia.^[11]^ During the 2003 severe acute respiratory syndrome epidemic, peripheral neuropathy was also reported.^[12]^

Olfactory function tests assess the degree of anosmia more objectively,^[13]^ and play an important role in the follow-up of patients to assess therapeutic response. In this case, follow-up was not performed because the patient preferred to remain at home to prevent spreading the infection.

On day 14 of the disease, the patient started taking vitamin B complex which has been used for peripheral neuropathy despite the lack of strong evidence for its benefits and its non-inclusion in treatment guidelines. Subsequently, the patient started an olfactory training program. Although the exact mechanisms of an olfactory training are unknown, based on animal and human research studies, we can assume that repeated exposure to an odorant may modulate the regenerative capacity of the olfactory receptors.^[14]^

Following this day, the patient continued improving until he recovered completely on day 30. It is impossible to assess whether our patient's clinical improvement was influenced by these interventions or whether the disease followed its natural course. However, considering the safety, low cost, and ease of access to olfactory training, in addition to the lack of clinical trials or well-designed studies, we believe olfactory training is a sound therapeutic option to be indicated in the clinical management of olfactory disorders in COVID-19 patients.

Additionally, it is important to emphasize that anosmia has several repercussions in an individual's life. As reported by previous studies, depending on the severity, it can compromise the quality of life and increase the mortality up to 4 times.^[4,15]^ The taste of food is strongly determined by the olfactory experience; consequently, the lack of a sense of smell worsens food perception and reduces appetite. Furthermore, the lack of perception of irritating smells such as gas, smoke, and volatile products is an important safety vulnerability factor. Moreover, many patients report difficulties with personal hygiene and excessive concern about body odor, bad breath, and problems with using perfumes. Interpersonal and sexual relationships can also be seriously affected. Finally, depending on the profession, an olfactory disorder may interfere or even prevent the performance of the affected individual.^[4]^

In conclusion, we believe that this case report will contribute to the increase in clinical suspicion of anosmia in COVID-19 patients, allowing for the isolation of the suspected patient, and consequently, aiding in controlling the pandemic. Furthermore, the details of the disease course and recovery of olfactory function can guide clinical management for cases of COVID-19 who experience anosmia.

## Author contributions

**Conceptualization:** Neisa Santos Carvalho Alves Pissurno, Anamaria Mello Miranda Paniago.

**Data curation:** Neisa Santos Carvalho Alves Pissurno, Anamaria Mello Miranda Paniago.

**Formal analysis:** Neisa Santos Carvalho Alves Pissurno.

**Investigation:** Gislene Garcia de Castro Lichs, Evelyn Jaqueline Lima dos Santos, Angelita Fernandes Druzian, Sandra Maria do Valle Leone de Oliveira.

**Methodology:** Neisa Santos Carvalho Alves Pissurno, Anamaria Mello Miranda Paniago.

**Project administration:** Anamaria Mello Miranda Paniago.

**Supervision:** Anamaria Mello Miranda Paniago.

**Writing – original draft:** Neisa Santos Carvalho Alves Pissurno, Anamaria Mello Miranda Paniago.

**Writing – review & editing:** Neisa Santos Carvalho Alves Pissurno, Sandra Maria do Valle Leone de Oliveira, Anamaria Mello Miranda Paniago.
